# Characterization and evaluation of a self-microemulsifying drug delivery system containing tectorigenin, an isoflavone with low aqueous solubility and poor permeability

**DOI:** 10.1080/10717544.2017.1284946

**Published:** 2017-03-10

**Authors:** Yunrong Zhang, Li He, Shanlan Yue, Qingting Huang, Yuhong Zhang, Junyi Yang

**Affiliations:** 1 West China School of Pharmacy, Sichuan University, Chengdu, China,; 2 Chengdu Women and Children’s Central Hospital, Chengdu, China, and; 3 Medical College of China Three Gorges University, Yichang, China

**Keywords:** Tectorigenin, self-microemulsifying drug delivery system, *in situ* gastric and intestine perfusion, bioavailability, enterohepatic circulation

## Abstract

The purpose of this study was to characterize and evaluate tectorigenin-loaded self-microemulsifying drug delivery system (TG-SMEDDS), a previously studied preparation, and further confirm the improvement of TG in solubility and bioavailability. The appearance of TG-SMEDDS was clear and transparent, with good mobility. The microemulsion formed by TG-SMEDDS was globular, edge smooth, clear-cut, and distribution homogeneous under transmission electron microscope. The stability studies revealed that TG-SMEDDS remained stable at room temperature for at least 3 months. TG-SMEDDS showed excellent dissolution behavior that more than 90% of TG was released in only 5 min. The *in situ* intestinal perfusion studies indicated enhancement of absorption in four tested intestinal segments, and the main absorption site of TG was changed to duodenum. In addition, TG-SMEDDS showed significantly higher *C*_max_ and *AUC* values (11-fold and 5-fold higher values, respectively; *P* < 0.05) than TG, and the absolute oral bioavailability of TG-SMEDDS was 56.33% (5-fold higher than that of crude TG). What’s more, the *AUC_0-t_* of crude TG and TG-SMEDDS in bile duct non-ligation rats were 6.05 and 2.80 times, respectively, than that in bile duct ligation rats, indicating the existence of enterohepatic circulation and the secretion of bile could significantly affect the absorption of TG. Further studies showed that even the bile duct was ligation, TG-SMEDDS can still keep a better oral bioavailability (179.67%, compared with crude TG in the bile duct non-ligation rats). Therefore, our study implies that SMEDDS containing TG could be an effective strategy for the oral administration of TG.

## Introduction

Tectorigenin (TG) is an isoflavone extracted from Belamcandae Rhizoma, the rhizome of the Chinese herb *Belamcanda chinensis* (L.) DC. TG has been proved to be one of the most active constituent in Belamcandae Rhizoma. A large amount of reports have indicated that it possesses a variety of pharmacologic actions, such as anti-inflammatory (Pan et al., 2008, Conforti et al., 2009), antibacterial (Ki-Bong Oh and Matsuoka, 2001), neutralize free radicals (Kang et al., 2005), selective estrogen receptor modulator (Shim et al., 2014), anti-tumor (Thelen et al., 2005, Yang et al., 2012), etc. The biopharmaceutical properties of TG had been investigated during pilot studies. The results indicated that TG belongs to Biopharmaceutics Classification System (BCS) class IV with low aqueous solubility and poor permeability, which challenges its clinical application. Hence appropriate approaches which enhance the solubility and permeability of TG were considered crucial to improve the oral bioavailability and to promote the clinical application.

In recent years, literatures about TG mainly focus on the pharmacological action and its mechanism. Previously in our laboratory, a series of TG preparations were studied including liposome, cyclodextrin inclusion complex, solid dispersion (Shuai Shuping et al., 2016), etc. After the preparation of TG into liposomes, the entrapment efficiency was too low to reach an appropriate dose. Cyclodextrin inclusion complex and solid dispersion increased the solubility of TG, which were, respectively, 8-fold and 30-fold higher than that of crude TG. Among them, the solid dispersion was better. However, it is not clear whether the dosage form can promote transmembrane absorption. Self-microemulsifying drug delivery system (SMEDDS) possesses the ability to simultaneously improve drug solubility and permeability, which has been increasingly employed to enhance the oral bioavailability of drugs (Bing Wang et al., 2015; Ding et al., 2016). SMEDDS is a mixture of oil, emulsifier, and co-emulsifier. The preparation can form transparent oil-in-water microemulsion when exposed to solvent media (such as gastrointestinal fluids) and stirred slightly. It is easy for SMEDDS to spread in the gastrointestinal tract (Qureshi et al., 2015; Yeom et al., 2015). Besides, the peristalsis of the intestinal tract can also provide stirring force for self-micro emulsifying. Thus, studies on the formula and preparation technology of a TG-loaded SMEDDS were conducted and completed in our previous researches. The formulation of the SMEDDS was as follows: 13% Capryol 90 (oil), 48% Cremophore RH40 (emulsifier), and 39% Transcutol HP (co-emulsifier). Preliminary results showed a significant increase in solubility. In the present study, TG-SMEDDS was systematically characterized and evaluated *in vitro, in situ*, and *in vivo*.

The rat *in situ* intestinal perfusion is a commonly used technique for the assessment of absorption and permeability of drugs, by measuring the disappearance of test substances from the perfused intestinal segment (Ho et al., 2008). The method provides an intact blood supply and the property of absorption determined by such method would be more consistent with the *in vivo* situation (Escribano et al., 2012). Thus, in this study, the absorption of TG-SMEDDS was evaluated in an *in situ* rat gastric and intestinal perfusion model.

The primary purposes of the present study, therefore, were to conduct systematic evaluation of TG-SMEDDS to investigate whether the solubility and oral bioavailability was improved compared with crude TG. At first, the appearance, morphology, and stability of TG-SMEDDS were characterized. Then *in vitro* dissolution tests of crude TG and TG-SMEDDS were employed to investigate the dissolution property. Finally, TG and TG-SMEDDS were further investigated to obtain the absorption properties and oral bioavailability in rats.

## Materials and methods

### Materials

TG reference substance was obtained from Must Bio-Technology Co., Ltd. (Chengdu, China), and the purity was ≥ 98%. TG test product and TG-SMEDDS were both laboratory self-prepared. Capryol 90 and Transcutol HP were all obtained from France Gattefosse Co. Cremophore RH40 was supplied by German Basf Inc. (Ludwigshafen, German). Phosphoric acid was purchased from Chengdu high tech Zone Shiyang Chemical Factory (Chengdu, China). Methanol of high-performance liquid chromatography (HPLC) grade was obtained from Honeywell Burdick & Jackson (Morristown, NJ). All other chemicals used were of analytical grade.

### Preparation

TG-SMEDDS was prepared as follows: Capryol 90 (13%), Cremophore RH40 (48%), and Transcutol HP (39%) were mixed by magnetic stirring at room temperature. After complete dissolution, SMEDDS, a clear and transparent solution, was obtained. Then, prescribed amount of crude TG was added into the SMEDDS (drug loading: 20 mg/g), continued stirring till the homogenous mixture was formed. Finally, a TG-loaded SMEDDS was obtained.

### Characterization

#### Appearance and morphology

A transmission electron microscope (TEM) was used to observe the morphology of TG-SMEDDS (Cui et al., 2009). At first, TG-SMEDDS was diluted with distilled water and mixed by slightly stirring. Then, applied an appropriate amount of the diluted TG-SMEDDS on a film-coated copper grid and stained it with 2% aqueous solution of phosphotungstic acid for 10 min, waiting to dry before observation under the transmission electron microscope.

#### Stability studies

In order to evaluate the stability of TG-SMEDDS, it was packed in sealed glass vials and then stored at room temperature for 1 month, 2 months, and 3 months. Samples were withdrawn at predetermined intervals and evaluated for parameters like physical appearance, concentration of TG, particle size after dilution with distilled water at 1:10, PDI, and viscosity. The stability studies were performed in triplicate for each condition. The concentration of TG was analyzed by HPLC method. The PDI and particle size after diluted was determined by using a Zetasizer nano laser particle size analyzer (Sun et al., 2011). Besides, the viscosity of TG-SMEDDS was determined by using a viscosimeter after dilution with distilled water at 1:10.

### *In vitro* dissolution tests

In order to study the dissolution characteristics of TG-SMEDDS and compare with the crude TG, the dissolution test was carried out and the Ch.P 2015 apparatus I (basket) method was used. TG-SMEDDS containing 5.5 mg of TG or 5.5 mg of crude TG was filled in size ‘0’ hard gelatin capsules. The revolution speed of the paddle was set as 100 rpm. About 1000 mL of hydrochloric acid solution (pH 1.2) and phosphate buffer (pH 6.8) were adopted as the dissolution mediums (Borhade et al., 2008; Setthacheewakul et al., 2010). And the temperature was kept at 37 °C ± 0.5 °C. In the course of the experiment, samples (5 mL) were obtained at predetermined sampling points (2, 4, 6, 8, 10, 15, 20, 25, 45, 60, 75, 90, 120, 150, 180, and 240 min) and the removed volume was replaced with fresh buffer. Then, the samples were filtered through a 0.45 μm polyvinylidene difluoride membrane and the concentration of TG was determined by HPLC.

### *In situ* gastrointestinal absorption evaluation in rats

*In situ* gastrointestinal absorption evaluation of both TG-SMEDDS and TG were performed and compared with each other. SD rats weighing 200 ± 20 g (SCXK (chuan) 2013-026) were provided by Experimental Animal Center of Sichuan University, China. All the rats were housed in an air-conditioned room under constant temperature (22 ± 2 °C) for at least 5 d with access to food and water. Before the experiments, they were fasted for 12 h with access to water.

#### Stability of TG in blank perfusates

The stability in blank perfusate was conducted to rule out the effect of stability factor on drug loss from the perfusates (Zhou Yue, 2006; Li et al., 2011). The stability of TG in blank gastric was performed by incubating TG gastric perfusate (20 μg/mL) and TG-SMEDDS gastric perfusate (20 μg/mL) in a water bath at 37 °C for 3 h, respectively. Samples were taken at 0, 1, 2, and 3 h. The stability of TG in blank intestinal perfusate was conducted in the same way and the sampling was performed at 0, 2, 4, and 6 h. The concentrations were determined by HPLC. The blank gastric perfusate, namely the simulated gastric fluid contained no pepsase, was prepared by diluting 16.4 mL of dilute hydrochloric acid to 1000 mL with water.

#### Effect of physical adsorption in gastric and intestine

The gastric and 10 cm of duodenum, jejunum, and ileum were removed, everted, washed, and ligated in both ends (Zhou Yue, 2006; Li et al., 2011; Yu et al., 2014). Then the harvested gastrics were respectively placed into crude TG gastric perfusate (20 μg/mL) and TG-SMEDDS gastric perfusate (20 μg/mL), followed by incubating in a water bath at 37 °C for 2 h. The TG or TG-SMEDDS gastric perfusate (20 μg/mL) were prepared by dissolving corresponding dose of TG or TG-SMEDDS into appropriate volume of blank gastric perfusate. Similarly, the harvested intestine segments were placed into crude TG intestinal perfusate (20 μg/mL) and TG-SMEDDS intestinal perfusate (20 μg/mL) respectively and incubated at 37 °C for 2 h. Finally, the concentrations at 0 h and 2 h were determined and the residual percentages of TG after incubation for 2 h were calculated.

#### The absorption in gastric

The absorption property in gastric was performed according to a previously described method in rats (Liu Taiming, 2006; Zhou Yue, 2006). In brief, rats were anesthetized by intraperitoneal injection of 20% urethane solution and then placed under an infrared lamp to maintain normal body temperature. Then, exposed the gastric surgically and cut a small opening in the pylorus. After that, the cardia was ligated and the artificial gastric juice was injected into the gastric through pylorus to wash the contents inside, until the cleaning fluid became clear. Subsequently, 4 mL of the gastric infusion containing TG or TG-loaded SMEDDS was injected into the gastric and then the pylorus was ligated immediately, followed by putting the gastric back into the abdominal cavity. The dose of TG in the gastric infusion was 20 μg/mL. After 2 h, the gastric was taken out and the drug fluid inside the gastric was transferred. A small amount of artificial gastric juice was used to wash the inside of the gastric and the washing fluid was combined with the drug fluid. Finally, the artificial gastric juice was used to adjust the volume of the above mixed fluid to 10 mL. The artificial gastric juice was simulated gastric juice contained no enzyme and was prepared by diluting 16.4 mL of dilute hydrochloric acid to 1000 mL with water. The drug concentration in the gastric perfusate was the initial concentration and the final concentration was the concentration of the drug in the final 10 mL of mixed fluid. The absorption percentage per hour (*P*%) in the gastric was calculated by the following equation: *P*% = (*C*_*0*_ × *V*_*0*_-*C*_*t*_ × *V*_*t*_) /( *C*_*0*_ × *V*_*0*_ × *t*) × 100%, where *C_0_* is the initial concentration of gastric perfusion fluid, *V*_0_ is the initial volume of gastric perfusion fluid, *t* is the time of gastric perfusion, *C_t_* is the final concentration of gastric perfusion fluid, *V_t_* is the final volume of gastric perfusion fluid.

#### The absorption in small intestine

*In situ* intestinal perfusion was carried out based on the previously established method in rats to investigate the absorption properties of TG-loaded SMEDDS and crude TG in duodenum, jejunum, ileum, and colon (Liu Taiming, 2006; Zhou Yue, 2006; Cui et al., 2009; Liu et al., 2009). Briefly, rats were anesthetized by intraperitoneal injection of 20% urethane solution, followed by fixed the back on the operating table with an infrared lamp to keep normal body temperature. Tested intestine was surgically exposed and isolated, and then approximately 10 cm of the intestine was cannulated with plastic tubing at both ends. The cannulated segment was attached to the perfusion assembly containing a constant flow peristaltic pump and was rinsed with 37 °C physiological saline to flush out the intestinal contents, until the outflow liquid became clear. Then, intestinal perfusion solution containing TG-SMEDDS or crude TG was perfused through the intestinal segment at a flow rate of 5 mL/min for 10 min to reach a steady state, followed by adjusting the flow rate to 2.5 mL/min. And at the same time, 1 mL of the perfusion solution was taken out and replaced by adding in 1 mL of blank phenol red solution, immediately. This time was recorded as 0 min and then sampling in the same way at predetermined time point of 15, 30, 45, 60, 90, 120, 150, and 180 min. Finally, the intestine segment was cut and the area was measured. Because the small intestine can absorb the water in the intestinal perfusate, the phenol red solution, which would not be absorbed by small intestine, was used to corrected volume of the intestinal perfusat. The concentration of TG in the TG-SMEDDS intestinal perfusion solution was 20 μg/mL, as well as the crude TG intestinal perfusate. All the samples were kept frozen at −40 °C until analysis. To analyze the samples, they were fused and centrifuged at 10 000 rpm for 5 min, and then 20 μL of supernatant was introduced to HPLC.

The intestinal perfusate was prepared by Krebs–Ringer's phosphate buffer solution and TG-loaded SMEDDS or crude TG. Krebs–Ringer's phosphate buffer solution contained 7.8 g NaCl, 0.35 g KC1, 1.37 g NaHCO_3_, 0.32 g NaH_2_PO_4_, 0.02 g MgC1_2_, 0.37 g CaCl_2_, and 1.40 g glucose in 1000 mL distilled water.

The average absorption percentage per unit area (*P*_s_%) was calculated by the following equation: *P*_s_% = (*C*_*0*_ × *V*_*0*_-*C*_*t*_ × *V*_*t*_)/( *C*_*0*_ × *V*_*0*_ × *S*) × 100%, and the average absorption rate per unit area (*P*%) was calculated by the following equation: *P*% = (*C*_*0*_ × *V*_*0*_-*C*_*t*_ × *V*_*t*_)/( *C*_*0*_ × *V*_*0*_ × t × *S*) × 100%. In the two equations, *C*_0_ is the initial concentration of intestinal perfusion fluid, *V*_0_ is the initial volume of intestinal perfusion fluid, *t* is the time of perfusion circulation, *C_t_* is the perfusion solution concentration at time *t, V_t_* is the volume of perfusion fluid at time *t, S* is the area of the tested intestinal segment.

### *In vivo* pharmacokinetics study

*In vivo* pharmacokinetic studies in bile duct non-ligation and bile duct ligation rats were performed in SD rats with 200 ± 20 g body weight. Before the experiment, rats were fasted overnight with free access to water.

TG suspension and TG-SMEDDS were given to rats (both 40 mg/kg of body weight) by intragastric administration. Blood samples were obtained at a volume of 250 μL by tail-cut sampling at the indicated times (0.033, 0.083, 0.167, 0.333, 0.5, 1, 2, 4, 6, 8, 12, 16, 24, 36, 48, and 60 h). Each blood sample (250 μL) was centrifuged at 8000 rpm for 5 min to obtain plasma samples. The obtained plasma samples were kept frozen at −40 °C until they were analyzed. TG concentrations in plasma were determined by HPLC. The method for sample pretreatment was as follows: briefly, 200 μL of precipitator (methanol:acetic acid, 9:1) was added to 100 μL of plasma sample, and the solution was vortex mixed for 10 min and centrifuged at 12 000 rpm for 5 min. Then 200 μL upper layer was transferred to another tube and evaporated at 40 °C by a nitrogen evaporator. The residue was reconstituted by 100 μL of mobile phase and vortex mixed for 3 min, followed by centrifuging at 12 000 rpm for 5 min. After that, 20 μL of the supernatant was injected for HPLC analysis.

*In vivo* pharmacokinetic study in bile duct ligation rats was performed to investigate the presence of enterohepatic circulation and the effect of bile on TG. The bile duct ligation model building method was as follows: rats were anesthetized and the dorsal position was fixed on the operating table. Infrared light was used to maintain normal body temperature and alcohol was adopted to wipe the surgical site for disinfection, followed by exposing the bile duct through operation. After that, the common bile duct was ligated, the tissue was restored; the peritoneum, the muscle layer, and the skin were successively sewed up. After the operation, penicillin was injected intraperitoneally to prevent infection and then resumed feeding for 5 d (Rivera-Espinosa et al., 2003; Horiuchi et al., 2009). Eventually, the pharmacokinetic tests started and the method was the same as the experiment of *in vivo* bioavailability study in bile duct non-ligation rats.

### High-performance liquid chromatography analysis

All compounds and internal standard were separated on a Kromasil C18 column (4.6 × 150 mm, 5 μm). The mobile phase consisted of methanol and water at pH 3.0 (60:40, v:v). The effluent was monitored at a UV absorption wavelength of 268 nm at a flow rate of 0.8 mL/min. The retention times of TG were 9.7 min. The calibration curve of TG was linear within the concentration range of 0.024–9.71 μg/mL, and the lower limit of qualification was 0.024 μg/mL. The intra-day and the inter-day precision (RSD) of TG were lower than 5.77%, and the recovery rates were up to 98.93%. All the data obtained in the method validation met the requirements of quantitative analysis. The maximum plasma concentration (*C*_max_) after oral administration and the time at which it was observed (*T*_max_) were observed directly from the individual plasma concentration–time profiles. Area-under-the-plasma concentration–time curve from 0 to 60 h (AUC_0–60_) was calculated by the linear trapezoid rule.

### Statistical analysis

Pharmacokinetic parameters were obtained using drug and statistics (DAS) version 2.1.1 software (Mathematical Pharmacology Professional Committee of China, Shanghai, China). Statistical differences among groups were assessed using the ANOVA test. When *p* < 0.05, statistical significance was considered to be achieved.

## Results

### Characterization of TG-SMEDDS

The appearance of TG-SMEDDS was homogeneous, transparent, yellow, liquid, and slightly sticky. The morphology of it was observed using a transmission electron microscope (TEM). When diluted with water, the microemulsion formed by TG-SMEDDS was globular, edge smooth, clear-cut, and distribution homogeneous under TEM.

The stability of TG-SMEDDS was investigated and the results are shown in Table 1. From the table, we can see that the appearance, content, particle size, and PDI of TG-SMEDDS showed no significant change after been long-term saved at room temperature even at the end of 3 months, indicating that the nature of the preparation was stable and the quality would not changed under the above conditions.

**Table 1.** Stability evaluation of TG-SMEDDS after been long-term saved (x±*s, n *=* *3).

Time (month)	Appearance	Content (%)	Particle size (nm)	PDI	Viscosity (mpa × s)
0	Yellow clear liquid	98.70 ± 0.31	14.80 ± 0.64	0.116 ± 0.009	2.15 ± 0.03
1	Yellow clear liquid	98.64 ± 0.67	14.91 ± 0.13	0.115 ± 0.004	2.14 ± 0.01
2	Yellow clear liquid	97.30 ± 0.25	15.07 ± 0.77	0.113 ± 0.010	2.17 ± 0.06
3	Yellow clear liquid	97.81 ± 0.20	14.98 ± 0.67	0.116 ± 0.014	2.18 ± 0.03

### *In vitro* dissolution tests

**Figure 1.**
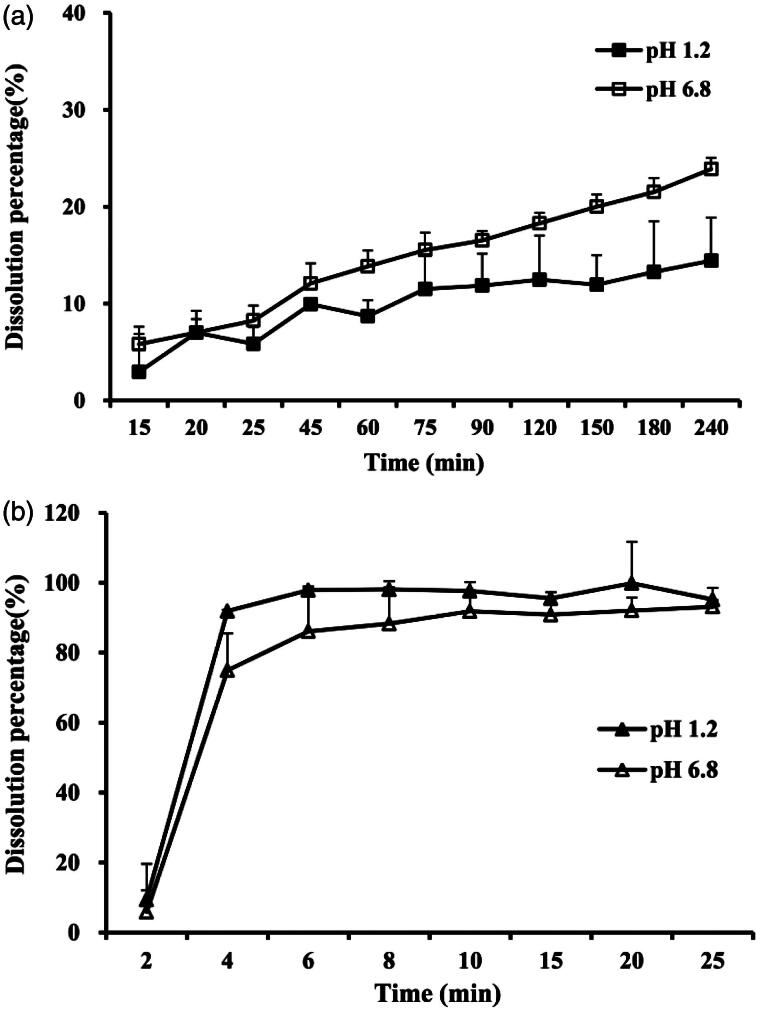
Dissolution profiles of crude TG in pH 1.2 (a, black square) or in pH 6.8 buffer solution (a, blank square), and TG-SMEDDS in pH 1.2 (b, black triangle) or in pH 6.8 buffer solution (b, blank triangle).

Dissolution profiles of TG-SMEDDS and crude TG powder were determined in two dissolution mediums (pH 1.2 and 6.8) and the results are shown in Figure 1. It can be seen from the figure that more than 90% of TG from SMEDDS was released within merely 5 min in both mediums (Figure 1b). Nevertheless, the crude TG showed low dissolution behavior that less than 20% was dissolved at 240 min (Figure 1a), which may be attributed to its poor solubility. The results indicated that the SMEDDS dramatically improved the dissolution of TG.

### *In situ* gastrointestinal absorption evaluation

#### Stability of TG in blank perfusates

In the stability studies of TG in blank gastric perfusates, the results showed that the concentrations of crude TG and TG from SMEDDS after 3 h were 20.07 ± 0.18 μg/mL and 20.00 ± 0.09 μg/mL, respectively. Moreover, the concentrations were 19.70 ± 0.47 μg/mL and 19.78 ± 0.11 μg/mL, respectively, in blank intestinal perfusates after 6 h. The above results indicated that no significant change of TG in concentration was found in neither blank gastric perfusates nor blank intestinal perfusates. We can conclude that both TG and TG-SMEDDS can keep stable in blank gastric perfusate for at least 3 h and in blank intestinal perfusate for at least 6 h, at the temperature of 37 °C. The results indicated that the stability factor had no impact on drug loss from the perfusates in our subsequent research (Zhou Yue, 2006).

#### Effect of physical adsorption in gastric and intestine

In adsorption studies, the residual percentages of crude TG and TG from SMEDDS in gastric perfusate were merely 73.08 ± 2.68% and 74.02 ± 1.54%, respectively, indicating that there was significant physical adsorption of TG in gastric and it should be taken into consideration in the experiment of *in situ* gastric absorption. Besides, the residual percentages of crude TG and TG from SMEDDS in intestine perfusate were approximately 100%, suggesting that no significant adsorption existed in tested intestine regions (Zhou Yue, 2006).

#### The absorption in gastric

The method of *in situ* gastric perfusion was used to investigate TG’s absorption characteristics in gastric. The hourly absorption percentages (*P*%) of TG-SMEDDS and crude TG in gastric are shown in Table 2. We have found that there was physical adsorption of TG on the gastric wall and *AMU* in Table 2 was the physically adsorbed rate which should be deducted from the parameter of *P*%. The adjusted hourly absorption percentage (*P*′%) of TG-SMEDDS and crude TG were 18.04% and 16.19%, respectively. *T*-test was used for the statistical comparisons among the gastric absorption of TG-SMEDDS and TG. The results indicated that there was no significant difference (*p* > 0.05) among the absorption of the two dosage forms in gastric. The possible reason might be that the gastric was not the main absorption site of TG.

**Table 2.** Absorption percentage per hour of TG in gastric perfusate (%, *n *=* *5).

Parameter	TG-SMEDDS	TG
*P %*	31.43 ± 0.30	29.65 ± 5.43
*AMU*	13.39 ± 1.59	13.46 ± 1.34
*P*′%	18.04	16.19

*AMU*, percentage of attachment, metabolism, uptaking by stomach per hour.

*P*′%, corrected absorption percentage per hour, *P*′% = *P*%-*AMU*.

#### The absorption in small intestine

The method of *in situ* intestinal perfusion was used to investigate the absorption in tested intestinal segments. The average absorption percentage per unit area (*P_s_*%) and average absorption rate per unit area (*P*%) of TG at four segments are listed in Table 3. Comparing with the results of crude TG, *P_s_*% of TG-SMEDDS were increased significantly from 1.65 ± 0.30% to 4.02 ± 0.37% in duodenum, from 1.23 ± 0.29% to 3.07 ± 0.30% in jejunum, and were also increased slightly from 3.21 ± 0.41% to 3.38 ± 0.40% in ileum, and from 2.16 ± 0.18% to 2.31 ± 0.08% in colon. The results revealed that the absorption extent of TG was enhanced by being prepared to TG-SMEDDS. Moreover, the *P*% of TG-SMEDDS in tested regions was also increased compared with crude TG, indicating the increasement of absorption speed in small intestine. Namely, both the speed and the extent of absorption in four different regions were enhanced when TG was prepared to TG-SMEDDS. The reason may be due to the increase of the solubility and transmembrane transport capacity of TG. Besides, the duodenum and jejunum provide the most efficient areas in the gastrointestinal tract for drug absorption. When TG was prepared into SMEDDS, the solubility increased and the area became the main factor affecting the absorption. Thus, the speed and were extent of absorption enhancement in duodenum and jejunum were more than those in ileum and colon.

**Table 3.** Absorption parameters of TG at intestinal segments (x±*s, n *=* *5).

	Duodenum		Jejunum		Ileum		Colon	
Perfusate	*P_*s*_*/%	*P* /%·cm^−2^	*P_*s*_*/%	*P*/ %·cm^−2^	*P_*s*_*/%	*P*/ %·cm^−2^	*P_*s*_*/%	*P*/ %·cm^−2^
TG	1.65 ± 0.30	0.55 ± 0.08	1.23 ± 0.29	0.41 ± 0.08	3.21 ± 0.41*	1.07 ± 0.18*	2.16 ± 0.18	0.72 ± 0.07
TG-SMEDDS	4.02 ± 0.37♦	1.34 ± 0.12♦	3.07 ± 0.30	1.10 ± 0.17	3.38 ± 0.40	1.13 ± 0.12	2.31 ± 0.08	0.77 ± 0.03

*p < 0.05, compared the two parameters of TG in ileum with those in other intestinal segments.

♦ *p* < 0.05, compared the two parameters of TG-SMEDDS in duodenum with those in other intestinal segments.

In addition, ANOVA test was employed to compare and analyze the two parameters of crude TG and TG-SMEDDS. For TG, the two parameters were significantly larger in ileum than those in the other intestinal segments (*p *<* *0.05). And there was no significant difference in duodenum and jejunum (*p *>* *0.05). Thus, the sort order of absorption rate of crude TG in rat intestine was as follows: ileum > colon > duodenum ≈ jejunum. For TG-SMEDDS, the parameters were significantly larger in duodenum than those in the others (*p *<* *0.05), and no significant difference existed in jejunum and ileum (*p *>* *0.05). Hence, the sort order of absorption rate of TG-SMEDDS in rat intestine was as follows: duodenum > jejunum ≈ ileum > colon. Above all, the main absorption site of TG was changed from ileum to duodenum when prepared to TG-SMEDDS, which would be more conducive to TG’s absorption.

It could be predicted from the above results and conclusions that TG-SMEDDS would show a better oral absorption behavior in the pharmacokinetic study compared with crude TG and further studies were needed to confirm it.

### *In vivo* pharmacokinetic study

#### *In vivo* pharmacokinetic study in rats

**Figure 2.**
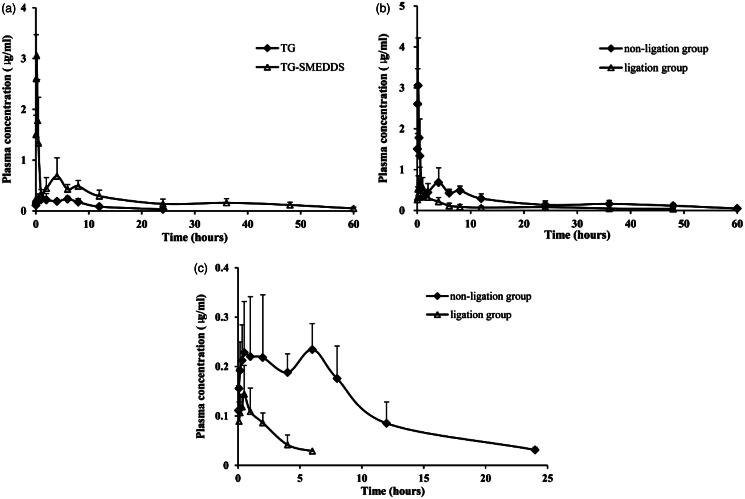
Mean plasma concentration–time profile of TG-SMEDDS/TG after orally administered to rats (a); mean plasma concentration-time profile of TG-SMEDDS after orally administered to non-ligation or ligation rats (b); mean plasma concentration–time profile of TG after orally administered to non-ligation or ligation rats (c).

The pharmacokinetic behaviors of crude TG and TG-loaded SMEDDS were investigated after oral administration in rats. Plasma concentration levels of TG were measured and plotted against time (Figure 2a).

During the period of 0–0.5 h, plasma concentrations of TG in rats receiving TG-SMEDDS were significantly higher than those receiving the crude TG. This initial high increment might due to the rapid dissolution induced by TG-SMEDDS. The pharmacokinetic parameters are listed in Table 4. Time needed to reach the maximum plasma concentration (*T*_max_) value of TG-SMEDDS was shorter than crude TG. TG-SMEDDS showed significantly higher maximum plasma concentration (*C*_max_) and *AUC* values (11-fold and 5-fold higher values, respectively; *p* < 0.05) than TG suspension. The enhancement could be attributed to the improvement in solubility, dissolution rate and permeability of TG from SMEDDS, since SMEDDS sequentially increased membrane fluidity and assisted in diffusion of the drug through the biological membrane.

**Table 4.** Statistical moment parameters of TG in rats after oral administration of TG/TG-SMEDDS in rats.

Parameters	TG	TG-SMEDDS	TG^a^	TG-SMEDDS^a^
*AUC_*0-t*_* (mg/L × h)	2.700 ± 1.012	13.588 ± 3.756♦	0.446 ± 0.174	4.851 ± 0.674
*AUC*_0-_*_*∞*_* (mg/L × h)	3.004 ± 0.985	15.437 ± 5.981♦	0.553 ± 0.312	5.422 ± 0.617
*MRT* (h)	6.98 ± 0.69	20.11 ± 2.36	3.31 ± 0.73	17.13 ± 3.99
*t_*1/2z*_* (h)	4.98 ± 1.52	18.68 ± 7.43	2.60 ± 1.64	13.84 ± 2.38
*T*_max_ (h)	1.00 ± 0.61	0.12 ± 0.05	0.70 ± 0.28	0.60 ± 0.22
	5.20 ± 1.79^b^	4.00 ± 2.45^b^		
*CLz/F* (L/h/kg)	14.34 ± 3.99	2.83 ± 0.79	91.30 ± 45.86	7.46 ± 0.93
*Vz/F* (L/kg)	99.90 ± 37.59	70.43 ± 17.20	284.06 ± 97.78	210.64 ± 131.56
*C*_max_ (mg/L)	0.310 ± 0.058	3.416 ± 0.985♦	0.154 ± 0.030	0.767 ± 0.257
	0.241 ± 0.040^b^	0.742 ± 0.322^b^		

^a^Bile duct ligation.

^b^ Parameter of another peak from double-peaks.

♦ *p* < 0.05, *C*_max_, and AUC values of TG-SMEDDS compared with those of TG.

The absolute oral bioavailabilities of TG and TG-SMEDDS were calculated using the equation: *F*_*abs*_ = ((*AUC*_*t*_ × *X*_*i.v*_)/(*AUC*_*i.v*_ × *X*_*t*_)) × 100%, where *F_abs_* is the absolute oral bioavailability, *AUC_t_* is the *AUC* of drug administrated orally, *AUC_i.v_* is the *AUC* of drug administrated by intravenous injection, *X_t_* is the dosage of oral administration, and *X_i.v_* is the intravenous injection dose. As a result, the absolute oral bioavailabilities of TG-SMEDDS were 56.33%, which was five-fold higher than that of crude TG (11.19%). The relative oral bioavailability of TG-SMEDDS was calculated by the equation: *F*_*rel*_ = ((*AUC*_*t*_ × *X*_*r*_)/( *AUC*_*r*_ × *X*_*t*_)) × 100%, where *F_rel_* is the relative oral bioavailability, *AUC_t_* is the *AUC* of TG-SMEDDS, *AUC_r_* is the *AUC* of TG, *X_t_* is the dosage of TG-SMEDDS, and *X_r_* is the dosage of TG. By calculating, the relative oral bioavailability of TG-SMEDDS was 503.26%. The data of results indicated that both the absolute and the relative oral bioavailability of TG in rats had been significantly improved by preparing it to TG-SMEDDS. Hence, TG-SMEDDS would become a promising formulation.

#### *In vivo* pharmacokinetic study in rats after bile duct ligation

Previously in the pharmacokinetic study, multi-peak phenomenon was found in the drug concentration–time curve, which may be related to the irregular absorption of drugs in the gastrointestinal tract or enterohepatic circulation (Chen et al., 2014; Kim et al., 2015). In order to study the existence of enterohepatic circulation, the pharmacokinetic behaviors of TG in bile duct ligation rats were investigated after oral administration of TG-SMEDDS and crude TG. Plasma concentration levels of TG were measured and plotted against time (Figure 2b and c).

The pharmacokinetic parameters after bile duct ligation are listed in Table 4. From the table, we could see that after ligation of the bile duct, maximum plasma concentration (*C*_max_) and *AUC_0−t_* were significantly reduced for both TG and TG-SMEDDS. The *AUC_0−t_* of crude TG and TG-SMEDDS in bile duct non-ligation rats were 6.05 and 2.80 times, respectively, than that in bile duct ligation rats, which showed that for TG-SMEDDS, the difference caused by bile duct ligation was not as significant as crude TG. In terms of the drug elimination, the *t*_1/2_*_z_* of crude TG was obviously decreased after bile duct ligation. And the *t*_1/2_*_z_* of TG-SMEDDS was also slightly reduced compared with that of the non-ligation group. Furthermore, in the ligation group, the *AUC_0-t_* of TG-SMEDDS was obviously higher than that of the crude TG in the non-ligation group, which indicated that even the bile duct was ligation (meaning the effects of bile and enterohepatic circulation were excluded), TG-SMEDDS could still improve the extent of absorption compared with crude TG.

Using the results of crude TG after oral administration in bile duct non-ligation rats as a reference, the relative bioavailability of TG and TG-SMEDDS in bile duct ligation rats were 16.52% and 179.67%, respectively, while the relative oral bioavailability of TG-SMEDDS in bile duct non-ligation rats was 503.26%. The results suggested that after blocking the excretion and secretion of bile, the absorption of drug by gastrointestinal tract was significantly reduced. So it could be concluded that there was enterohepatic circulation in the process of TG absorption in rats’ gastrointestinal tract and the secretion of bile could significantly affect the absorption of TG. In addition, after oral administration of TG-SMEDDS to bile duct ligation rats, the relative bioavailability was 179.67%, indicated that after blocking the excretion and secretion of bile, the absorption of TG in TG-SMEDDS by the gastrointestinal tract was not affected and still increased, compared with the crude TG. The results and conclusions suggested that TG-SMEDDS has a great advantage in the absorption and bioavailability of TG, compared with crude TG.

## Discussion and conclusion

The therapeutic efficiency of TG is limited by its poor water solubility and low permeability after oral delivery. In this study, a new dosage form, SMEDDS, was applied to improve the solubility and the oral absorption of TG. At first, characterization of TG-SMEDDS was conducted and the results provided data support for the feasibility of pharmacokinetics of TG-SMEDDS in rats. The particle size of the preparation after microemulsification was less than 50 nm. It can be expected that TG-SMEDDS can form nano grade microemulsion after entering the gastrointestinal tract and exposing to gastrointestinal fluid, which will make the process of being absorbed into the body circulation through the intestinal epithelium more easily than crude TG (Kang et al., 2004; Wu et al., 2006; Setthacheewakul et al., 2010; Sermkaew et al., 2013). And thus the oral absorption be promoted and bioavailability be improved.

The method of *in situ* gastric perfusion and intestine absorption evaluation was used to investigate absorption properties of TG in SMEDDS. The research results showed that preparing TG into the SMEDDS significantly increased the absorption of TG in tested segments. Furthermore, after TG was prepared into SMEDDS, the main absorption site in the intestinal tract of rats was changed from ileum to duodenum. There are a number of structures of villi and annular folds in the duodenum, which make it possess a greater absorption area compared with other intestinal segments and be the better absorption site. In the experiment, to reduce the effect of intestinal absorption of water on the results, phenol red was added into the sample solution as a marker due to its extremely low absorption in the intestine (Liu et al., 2009).

As the results of the pharmacokinetics studies showed, the oral absorption and bioavailability of TG, a drug with poor solubility and low permeability, was enhanced by SMEDDS. The possible reasons were shown as follows: (1) the preparation of SMEDDS improved the solubility of TG (Bali et al., 2011): as previous researches in our laboratory showed, the solubility of TG was 44 mg/mL after preparing it to TG-loaded SMEDDS, much larger than that of crude TG(31.4 μg/mL). (2) The nano particle size of the preparation makes it more easily to diffuse into the mucosal layer: the results of particle size measurement showed that the particle size of TG-SMEDDS was only nanometer, and literatures reported that preparation of this particle size was more easily to be ingested through the mucosal layer (Kang et al., 2004; Wu et al., 2006; Sermkaew et al., 2013). *In situ* gastrointestinal absorption tests also confirmed the increasement of TG absorption by gastrointestinal. (3) Surfactant promoted the absorption (Spernath et al., 2002; Wong et al., 2006; Kishimoto et al., 2016): TG-SMEDDS contains a surfactant, which can damage the phospholipid bilayer of epithelial cells and is often used as a absorption enhancer. (4) Inhibition of CYP3A enzyme or P-gp: emulsifier Cremophor 40 has been proved to be a double inhibitor of P-gp and CYP3A enzyme (Bravo Gonzalez et al., 2004; Yamagata et al., 2007) and TG is a kind of flavonoid compound, which is a P-gp substrate. Therefore, TG-SMEDDS may inhibit the efflux of TG by P-gp, thereby improving the blood concentration of it.

In the pharmaceutics studies of bile duct ligation rats, the double-peak phenomenon was disappeared, indicating the existence of enterohepatic circulation and revealing the reason of double-peak phenomenon. The mechanism was as follows: drugs absorbed into the blood can be excreted into duodenum by the bile duct after liver metabolism and then some of them can be absorbed in blood again through the intestinal mucosa, resulting in second plasma concentration peak. The study also revealed that bile secretion can significantly affect the pharmacokinetics of TG. This kind of property may cause different reactions among patients when apply TG to clinic. For example, there may be a risk of significant reduction of drug efficacy in patients with jaundice and liver transplant. In addition, food intake may also affect the absorption of drugs. However, the relative bioavailability of TG-SMEDDS was 179.67% after bile duct ligation, revealing that the degree of TG-SMEDDS absorption was significantly better compared with crude TG, even the bile duct was tied. Namely, SMEDDS can reduce the effect of bile duct ligation on TG absorption. The present study mainly focused on blocking bile secretion to investigate the effect of bile. In the future study, the pharmacokinetics of orally deliver TG or TG-SMEDDS combined with bile acids can be conducted in bile duct ligation rats to further investigate the effect of bile on the absorption of TG. What’s more, the mechanism of reducing the effect of bile duct ligation by SMEDDS can be further researched.

In conclusion, a SMEDDS formulation for TG was characterized and evaluated in this study. The appearance was clear and transparent, yellow, with good mobility, and it showed a good stability. Dissolution percentage of TG in SMEDDS was significantly higher than that of crude TG. The absorption of TG from SMEDDS was obviously increased and the major absorption site was changed to duodenum. *In vivo* bioavailability studies suggested that both the absolute and the relative oral bioavailability of TG in rats had been significantly improved. Furthermore, the bioavailability study of bile duct ligation rats suggested the presence of enterohepatic circulation, explained the double-peak phenomenon and preliminarily showed that bile can promote the absorption of TG. Above all, the TG-SMEDDS in this study increased the dissolution rate and improved the oral bioavailability of TG. The study implies that SMEDDS could be a promising strategy for oral delivery of TG, and also provides reference for other drugs with poor aqueous solubility.
